# Cytokine release syndrome and CAR T Cell therapy: Modulating the intensity of the inflammatory response and resolution within the tumor microenvironment

**DOI:** 10.3389/fphar.2025.1615526

**Published:** 2025-06-10

**Authors:** David F. Driscoll, Bruce R. Bistrian

**Affiliations:** ^1^ Stable Solutions LLC, Easton, MA, United States; ^2^ Department of Medicine, UMASS Chan Medical School, Worcester, MA, United States; ^3^ Department of Medicine, Harvard Medical School, Boston, MA, United States

**Keywords:** CAR T cell therapy, cytokine release syndrome, inflammation, resolution, tumor microenvironment

## Abstract

CAR T cell therapy achieves high degrees of success with respect to complete response and overall response rates in many hematological cancers, especially lymphomas. Compared to other immunotherapies, these “activated” blood products are plagued by a high incidence of a severe systemic inflammatory response syndrome, resulting from the exaggerated release of cytokines, chemokines, and other pro-inflammatory protein and lipid mediators. These can produce what is known as the “cytokine release syndrome” (CRS), associated with significant morbidity and mortality. Although successful CAR T cell therapy reduces the tumor load, the killing of large numbers of cancer cells and the persistence of apoptotic cellular debris within the tumor microenvironment (TME) may also be tumorigenic. We propose a single active pharmaceutical ingredient (API), the highly polyunsaturated omega-3 fatty acids eicosapentaenoic and docosahexaenoic acids, applying a refined and enriched fish oil, with multiple therapeutic targets that can be administered in precise doses. First, they rapidly modulate the intensity of the systemic inflammatory response, by modifying eicosanoid metabolism via intravenous administration. Second, as substrates for the production of specialized pro-resolving mediators (SPMs) of inflammation, they can help clear cellular debris within the TME, perhaps reducing the risks of new tumor formation. The employment of such a drug either in a prophylactic and/or a treatment manner might further improve the outcome of CAR T cell therapy.

## Introduction

CAR T cell therapy has been shown to be highly effective in many hematological cancers, especially in B cell lymphomas, B cell acute lymphoblastic leukemia, and multiple myeloma (Cappell and Kochenderfer, 2023). Among the newest promising cancer immunotherapies, i.e., CAR T cell, bispecific T cell engagers and immune checkpoint inhibitors (Shah et al., 2023), CAR T cell therapies are truly novel, as they are derived by harvesting T-cells from the patient. Consequently, this approach has been referred to as a “living drug” therapy (De Marco et al., 2023). One of the earliest clinical reports of this novel therapy came from the National Cancer Institute at the NIH (Kochenderfer et al., 2010). Since 2017, no fewer than seven CAR T cell products have been approved by the FDA, with the latest approval for obecabtagene autoleucel (Aucatzyl^®^) on 8 November 2024. Unfortunately, CAR T cell therapy is not very effective in solid tumors due to several factors (antigen heterogeneity, infiltration problems, cell survival in the TME, off-target toxicity) (Chen et al., 2022; Zhao et al., 2025). To overcome this limitation, a novel approach includes macrophages engineered to express chimeric antigen receptors (i.e., CAR macrophages) that clear debris within the TME via phagocytosis and efferocytosis (Koppers et al., 2025), but significant gaps currently exist regarding CAR constructs, efficacy and safety (Li et al., 2024).

### CAR T Cell therapy and CRS

Of all existing immunotherapies, CAR T cell therapy is most frequently associated with the development of CRS. Like cytokine storm in critically ill patients with, for example, COVID-19 infection or hemaphagocytic lymphohistiocytosis (Fajgenbaum and June 2020; Henter J, 2025; Long JP et al., 2025), CRS is a consequence of an exaggerated systemic inflammatory response. Depending upon the trigger, it involves the outpouring of selected, high blood concentrations, of cytokines (Santurio et al., 2025), such as interleukin-1 or IL-1, IL-6, IL-10, and tumor necrosis factor or TNF. It also involves chemokines, such as IL-8, monocyte chemoattractant protein-1, or MCP-1, and macrophage inflammatory protein 1β, or MIP-1β. Finally, it results in the production of SPMs, from dietary intake of polyunsaturated fatty acids such as from arachidonic acid, i.e., Lipoxins; Resolvin E series, i.e., RvE1-4, from eicosapentaenoic acid; and, Resolvin D series, i.e., RvD1-6, from docosahexaenoic acid, as well as protectins and maresins (Kiyasu et al., 2024).

Moreover, CAR T cell therapy is also a labor intensive and expensive process (Sainatham et al., 2024), in which the patient’s blood is externally processed (by leukapheresis, genetically altered via CAR-encoding) and then replicated in the laboratory (*ex vivo* expansion) to generate a suitable “living dose”, and ultimately reinfused into the patient. Typically these cells will continue expansion *in vivo* post-infusion, generally climaxing within days after infusion, a time period that correlates with the onset of CRS (Lionel and Neelapu, 2024). Major risk factors for the development of CRS during CAR T cell therapy include the CAR product (the co-stimulatory domain in the CAR construct and the target antigen of the CAR), *ex vivo* processing/culturing parameters, and *ex vivo* T-cell selection processes, as well as tumor biology and burden/load (Hughes et al., 2024).

### Systemic inflammatory response (SIR)

The development of a systemic inflammatory response is generally viewed as beneficial to normally nourished patients during severe metabolic, infectious, and/or traumatic, stress. Early study of the pathogenesis of fever, a prime component of systemic inflammation, described a newly identified pro-inflammatory substance, or “endogenous pyrogen” (EP), which appeared to be derived from polymorphonuclear leukocytes (Atkins and Wood, 1955; King and Wood, 1958). Subsequently, this substance was referred to as leukocyte endogenous mediator (LEM), and this crude extract was able to produce fever as well as all of the other major components of the systemic inflammatory response. In critically ill, severely protein-malnourished patients a reduced capacity to produce LEM was noted (Keenan et al., 1982). In contrast, in a population of mild to moderately malnourished patients undergoing elective surgery, the ability to produce LEM was retained both before and after surgery, suggesting a “high biologic priority” for persistence of this ability in this population with better nutritional status and less inflammatory stress (Duncan et al., 1984). This suggested that the degree of malnutrition and metabolic stress may play significant roles in immune function. Subsequently, two major contributors (Dinarello and Cerami) to this field (Dinarello, 1984; Dinarello and Savage, 1989; Dinarello et al., 1990; Beutler and Cerami, 1985; Cerami, 1993; Tracey and Cerami, 1993) identified the two primary purified cytokines responsible for the systemic inflammatory response due to EP and LEM, i.e., IL-1 and TNF, respectively.

### Activation of the SIR

Consequently, the concept of activation of a systemic inflammatory response syndrome (SIRS) was formally developed to harmonize the definitions of the clinical response to sepsis (Bone et al., 1992). But it also applies to any severe metabolic stress encountered during non-infectious critical care conditions, e.g., burn injury, pancreatitis, multiple trauma, or other highly catabolic insults (Balk, 2014; Cabrera-Rivera et al., 2022). Furthermore, systemic inflammation is intimately involved in the acute phase response, such as increased mobilization of amino acids from skeletal muscle for the synthesis of acute phase proteins (Pomposelli et al., 1988; Bistrian, 1999). The acute phase proteins are largely produced by hepatocytes, but they are also generated by other cells that produce cytokines, such as organ-infiltrating monocytes, resident macrophages and Kupffer cells (Mantovani and Garlanda, 2023).

Of the many pro-inflammatory mediators secreted during CRS in patients receiving CAR T cell therapy, interleukin-6 (IL-6) appears to be the most prominent, and is secreted by activated endothelial cells (Levstek et al., 2024). As such, when CRS symptoms persist and/or are severe, first-line treatment with the IL-6 receptor antagonist tocilizumab, with or without corticosteroids, is indicated. Additional agents for treatment of CRS include another IL-6 antagonist siltuximab, or alternatively anakinra (IL-1 antagonist), but unfortunately the administration of these agents is associated with an increased risk of opportunistic infections, gastrointestinal perforation and anaphylactic reactions (Driscoll and Bistrian, 2024a). Other inflammatory biomarkers have been studied such as the CAR-HEMATOX risk score, Inflammation-Based Prognostic Score (IPBS), Endothelial Activation and Stress Index (EASIX), and Cumulative Illness Rating Score (CIRS), but none of these have proved conclusive (Levstek et al., 2024).

## Changes in the tumor microenvironment (TME)

Importantly, macrophages play a major role in responding to CAR T cell therapy within the TME resulting from a high tumor cell kill. Thus, successful CAR T cell therapy prompts apoptosis and rupture of cancer cell membranes within the TME, prolonging the localized inflammatory response. Over time the actions of macrophages will adapt to the TME (Ammarah et al., 2024) and thus, can be altered (polarized) within the TME from suppressing tumor growth, to promoting tumorigenesis, thus exhibiting a dual role, and inflammation is a key driver of this transformation (Morana et al., 2022; Liu et al., 2022; Park et al., 2023). In other cases, macrophages may suppress anti-tumor immune responses such as in B-cell Non-Hodgkin’s Lymphoma (Gao, 2025). The continued presence of unresolved “cellular debris” from macrophages persists in promoting local inflammation and cytokine release, which appears to enhance tumor progression activities.

### Therapeutic options to address the TME

Consequently, recognition of the transformation of macrophages from tumor suppression to tumor progression has increased the interest in preventing polarization of macrophages. Given the complexity of this process, multiple strategies and potential drugs have been identified, but unfortunately treatment involves several drugs or combinations thereof (Liu et al., 2022; Mantovani et al., 2022; Zhao et al., 2023; Yang et al., 2024; Cao and Liu, 2024). Not only do most of these compounds involve single targets, but the clinical ramifications of the proposed agents may have untoward drug/metabolic effects that may complicate, or even worsen, patient outcomes. An alternative, and possibly safer, approach includes the use of lipid autacoids, or SPMs that stimulate macrophages to phagocytize cellular debris within the TME and may also enhance the efficacy of immunotherapy (Sulciner et al., 2018; Chiang and Serhan, 2020; Lavy et al., 2021; Fishbein et al., 2021; Julliard et al., 2022; Serhan and Sulciner, 2023; Kiyasu et al., 2024; Toledo et al., 2024 Long et al., 2025).

However, if given as single injections or mixtures of SPMs, which agent or combination thereof would best suit the patient? As above, these are unanswered questions, as well as entailing unknown risks. Moreover, there are significant stability issues associated with SPMs that limit their usefulness in the clinical setting (Kiyasu et al., 2024; Maliha et al., 2024; Quinlivan et al., 2024). As well, in either case above, how does one calibrate the dose of each and titrate the response? Finally, although CAR T cell, exosome-based nanoparticles may be able to reduce the incidence of tumorigenesis, there are significant shortcomings. Their development is expensive and long-term storage can affect physical stability, exosome heterogeneity and loading problems, which present major pharmaceutical development issues (Ye et al., 2024; Zhang et al., 2024). Moreover, at present, there is no formal guidance (i.e., pharmacopeial-approved methods) to determine the physical stability of these nanoparticle formulations (Driscoll and Bistrian, 2024b).

### Polyunsaturated fatty acids and eicosanoid metabolism

Eicosanoids are a group of autacoid hormones derived from highly polyunsaturated compounds containing 20 and 22 carbon fatty acids. They are present in the diet as long-chain omega-6 essential fatty acids, arachidonic acid or ARA (20:4n6), derived from an 18-carbon precursor, i.e., linoleic acid (18:2n6) found in vegetable oil, such as soybean oil. Similarly, soybean oil also contains an 18-carbon precursor for omega-3 fatty acids (α-linolenic acid, 18:3n3), but unlike 18:2n6, its bioconversion to active forms in humans is extremely low (Arterburn et al., 2006), particularly distally to docosahexaenoic acid. Alternatively, the essential omega-3 fatty acids are also lipid substrates from the diet that principally include eicosapentaenoic acid or EPA (20:5n3) and docosahexaenoic acid or DHA (22:6n3), found in marine sources such as fish oil, including small amounts of ARA (≤4% by weight of the fatty acid profile). These fatty acids are key glycerophospholipid components of plasma cell membranes, such as white and red blood cells, and platelets, esterified in the *sn*-1 and *sn*-2 (stereospecific numbering positions), while *sn*-3 is a phosphodiester.

During high metabolic stress, the essential fatty acids are released from the cell membranes by the enzyme phospholipase A_2_, or PLA_2_. There appears to be a specificity of the enzymes for each essential fatty acid, with cytosolic PLA_2_ having a preference for ARA, and calcium-independent PLA_2_ having an affinity for EPA, and secreted PLA_2_ showing a predilection for DHA (Hayashi et al., 2021). The essential fatty acids play a key role in inflammation and immune function, forming ubiquitous second messengers (eicosanoids) via cyclooxygenase or COX enzymes that generate prostaglandins, thromboxanes and prostacyclins. In addition, lipoxygenase or LOX enzymes form leukotrienes, such as leukotriene B4 or LTB4, mainly generated by polymorphonuclear neutrophils, whereas, for example, leukotriene C4 or LTC4 is largely produced by eosinophils.

### Two tiered, multi-targeted approach

Historically, the dietary intake of omega-6 (or n6) compared to omega-3 (or n3) fatty acids in humans before the Industrial Revolution were equivalent, at a ratio of approximately 1:1, but today it is >15:1 (Simopoulis, 2008). Consequently, current patients in western civilization are primed towards having a more pro-inflammatory response to metabolic stress. Thus, omega-6 fatty acids will form the more vasoactive/pro-inflammatory 2-series prostaglandins, thromboxanes and prostacyclins, and the 4-series leukotrienes. In contrast, omega-3 fatty acids produce the less vasoactive/anti-inflammatory three series and 5-series prostanoids, respectively. Importantly, however, of all the major classes of lipids in the diet, that also includes omega-9 fatty acids, a “neutral” lipid (i.e., oleic acid/olive oil), omega-3 fatty acids are the preferred substrate (Bistrian, 2003). Consequently, acute intravenous supplementation with EPA and DHA will rapidly be incorporated into plasma cell membranes (Carpentier et al., 2010), producing less vasoactive secondary messengers with a resultant modulation (reduction) of the intensity of the systemic inflammatory response, despite the overabundance of omega-6 fatty acids in the body.

In addition to reducing systemic inflammation, there is a second valuable and major pharmacological action/benefit associated with polyunsaturated fatty acids such as ARA (i.e., lipoxins), and from EPA and DHA (i.e., resolvins, maresins, and protectins), that serve as substrates for the production of SPMs. In 2008, Serhan et al. first described in great detail that the conventional thinking that resolution was passive was incorrect, when indeed it was an active process in order to protect organs and tissues from “collateral damage” (Serhan et al., 2008). Since then, Serhan et al. have identified 6 classes of SPMs and subsets, including, for example, Class I: E-series Resolvins, or RvE (derived from EPA), RvE1, RvE2, RvE3 and Rv4; Class II: D-series Resolvins, or RvD (derived from DHA), RvD1, RvD2, RvD3, RvD4 and RvD5; Class III: Protectins, or D1 (derived from DHA), PD1 and Neuroprotectin D1, or NPD1, among others (Chiang and Serhan, 2020).

## Omega-3 fatty acid pharmacologic therapy in CRS and within the TME

### Therapeutic goals

There are two principal and potentially valuable therapeutic goals of providing omega-3 fatty acids to mitigate the consequences of CAR T cell therapy. First, they should be able to provide sufficient substrate for the cyclooxygenase and lipoxygenase enzymes to modulate the intensity of the hyper-inflammatory response associated with CRS by the production of less vasoactive mediators (e.g., prostaglandins, leukotrienes) within the plasma cell membranes of myeloid cells, thereby reducing cytokine and chemokine secretion. Second, they should be dosed to deliver the key substrates for SPMs to ensure resolution of apoptotic cells following successful CAR T cell therapy (high tumor cell kill). Compared to CAR macrophages, SPMs are a safer and currently available approach to promote clearance (phagocytosis and efferocytosis) of cellular debris within the TME (Quinlivan et al., 2024). A refined and enriched fish oil containing high concentrations of EPA and DHA, comprising more than 60% of the fatty acids by weight of the fatty acid profile (Driscoll et al., 2009), will stimulate the production of SPMs such as resolvins, protectins and maresins, as well as sufficient amounts of ARA for lipoxins.

### Bioavailability

From the outset, the only viable way to quickly provide the key polyunsaturated fatty acids, which serve as substrates for modulating eicosanoid metabolism and adequate production of SPMs, is by intravenous administration. Oral routes of administration of natural fish oil supplements take 6–8 weeks for high dose capsules in order for efficient incorporation into the plasma cell membranes (Endres et al., 1989), whereas with continuous enteral tube feeding it can be achieved within 5–7 days (Kenler et al., 1996). However, neither route of delivery would be fast enough to prevent or minimize the acute intensity of the inflammatory response associated with CRS, and now this issue also appears to be true as demonstrated in a recent large retrospective cohort of hospitalized patients with sepsis (Narayan et al., 2025), and in the accompanying editorial (Giamarellos-Bourboulis, 2025). In contrast, uptake of EPA and DHA by intravenous delivery occurs within hours of the infusion, particularly when accompanied by medium-chain triglycerides (Hamilton et al., 1996).

### Pharmaceutical criteria

At this time the only 100% fish oil (“refined-only”) emulsion suitable for intravenous use that is commercially available is a nutritional product known as Omegaven^®^, but as such, the concentrations of EPA and DHA in this natural fish oil product are highly variable (±50%) (Driscoll and Bistrian, 2023). There is another nutritional lipid injectable emulsion that also contains this refined-only fish oil, but it is a minor component in a 4-oil mixture known as SMOFlipid^®^, (containing, by weight: 30% Soybean oil, 30% Medium chain triglycerides or MCT, 25% Olive oil and 15% Fish oil). Given the high variability in the contents of EPA and DHA in these formulations, precise dosing is not achievable. In addition, there is also a commercial nutritional product that uses the “refined and enriched” fish oil known as Lipoplus^®^ or Lipidem^®^, but it too is a multi-oil mixture containing, by weight: 40% Soybean oil, 50% MCT oil, and 10% fish oil. Despite the fact there is more fish oil by weight (15%) in the refined-only mixture than the refined and enriched fish oil by weight (10%), the latter contains approximately 50% higher concentrations of EPA and DHA (Driscoll et al., 2008; Driscoll et al., 2009). Although the quality of this fish oil may also provide precise dosing of EPA and DHA, the amounts currently present in commercial formulations are likely too low to have a significant impact on acute systemic inflammation and/or resolution with the TME.

In the case of fish oil, the European Pharmacoeia (EP) has two separate monographs that are suitable for intravenous injection. One is a “refined-only” source designed for nutritional products (as described above), entitled “Fish Oil, Rich in Omega-3 Acids” (EP monograph no. 1912) and the minimum sum of EPA and DHA is 22% by weight of the fatty acid profile. Another fish oil for intravenous use that can deliver precise doses of EPA and DHA (±10%), is both refined and enriched. It is entitled “Omega-3 Acid Triglycerides” (EP monograph no. 1352), for which the minimum sum of EPA and DHA is 45% by weight. Using the EP 1352-based fish oil, a formulation meeting pharmacopeial specifications for an injectable emulsion containing 90% (by weight) of the refined and enriched fish oil, along with 10% by weight, of MCT oil was suggested (Driscoll and Bistrian, 2024a). Although both the refined-only, and refined and enriched fish oil sources are approved as official pharmacopeial articles (intended for intravenous use in humans), there is no other commercially available product that employs the ideal fish oil composition as described in EP 1352, containing higher than 10% (by weight as described). We believe this is a major shortcoming in the field and that providing such a product delivering precise pharmacological doses of the API (EPA + DHA) for use in intravenous administration could be readily made and rapidly titrated to achieve the desired clinical response.

### Potential formulation

In further pursuit of this hypothesis-generating model, we propose a formulation in Table 1 adhering to pharmacopeial specifications as a continuous intravenous infusion, based on an upper human dose limit of 6 g of EPA and DHA per day established from current nutritional intakes in the clinical setting (Driscoll and Bistrian, 2023), based on tumor load (Driscoll and Bistrian, 2024b). The therapeutic rationale for the formulation is to ensure a safe formulation, and one that can be infused at low rates that does not produce hypertriglyceridemia. Increasing the hydrocarbon chain length of the fatty acid triglyceride, for example, 8–10C → 18C → 20C → 22C, decreases the rate of clearance from the bloodstream and increases the incidence of infusion rate complications, i.e., fat overload, hypertriglyceridemia and interference with the immunological processes (Driscoll, 2017; Driscoll, 2023) of the reticuloendothelial system or RES (Saba TM, 1970).

**TABLE 1:** Proposed intravenous formulation^a^ ^,^ ^b^ to deliver precise amounts of EPA and DHA^c^.

Ingredient	Role	Amount/100 mL
Omega-3 acid triglycerides, EP no. 1352	Active pharmaceutical ingredient	18.0 g^d^ ^,^ ^e^
Medium Chain Triglycerides, EP no. 0868	Carrier/Stabilizer/Facilitates plasma clearance	2.0 g
Egg phospholipids, EP no. 2315E	Surfactant	1.2 g
Sodium oleate^f^	Co-surfactant	0.03 g
Glycerin, EP no. 0496	Tonicity agent^g^	2.5 g
α-Tocopherol, EP no. 0692	Antioxidant	0.02 g
Sodium hydroxide	pH adjustment (6.0–9.0^h^)	≤0.1 mmol/L
Water for injection, EP no. 0169	Aqueous Solvent/Vehicle for oil-in-water emulsion	*Quantum satis ad* 100 mL

^a^ Lipomega-3^™^.

^b^ stable for 18 months.

^c^ underwent safety review by the FDA.

^d^ supplies 10.8 g (±10%) of EPA, and DHA/100 mL providing about 100 ± 10 mg/mL of EPA/DHA, in a ratio of 1.5:1.

^e^ EPA + DHA, dose: 0.03–0.06 g/kg/day, up to 6 g/day by continuous intravenous infusion.

^f^ As described in United States Pharmacopeia or USP monograph entitled Lipid Injectable Emulsion, suitable stabilizer such as a fatty acid salt.

^g^ osmotic agent yielding a final product between 280–320 mOsm/L, 1.7%–2.5% by weight.

^h^ between 6.0–9.0.

We would also propose that this formulation and infusion schedule be given prophylactically after receipt of CAR T cell therapy. Of course, the ideal time frame and whether a small bolus dose should be administered prior to its continuous infusion, as well as other clinical considerations (timing, optimized dose and response, length of infusion, monitoring parameters, etc.), would have to be determined during experimental study in patients susceptible to CRS.

### CRS treatments

Presently, management of CRS is guided by a 4-point gradation algorithm based on the severity of symptoms, from mild to severe (Grade 1–4). Current medical management includes tocilizumab injection as the first-line agent either by itself, but can also be given along with intravenous anti-inflammatory corticosteroids (dexamethasone, methylprednisolone). After dose escalation without an adequate response, alternative anti-cytokine agents (i.e., siltuximab, anakinra) can be used. Proposed medical management of CRS provides a multi-targeted single API (EPA + DHA) in a weight-based dose (g/kg), based on tumor load (low: 0.03 g/kg/day; high: 0.06 g/kg/day), up to 6 g/day. Table 2 provides a pharmacotherapeutic comparison of the two drug regimens.

**TABLE 2:** Pharmacotherapy for CRS: *Single vs Multiple Target* ***s***.

*Current* Drug Therapies	Pharmacological Action (s)	*Proposed* Drug Therapy	Pharmacological Actions
Tocilizumab	IL-6 receptor antagonist	EPA + DHA	Formation of 3- and 5-series prostanoids and other lipid mediators; ↓secretion of cytokines, chemokines and other protein mediators; substrate for SPMs
Dexamethasone	Anti-inflammatory		
Methylprednisolone	Anti-inflammatory		
Siluximab	IL-6 receptor antagonist		
Anakinra	IL-1 receptor antagonist		

## Summary

We recognize that this proposal of a novel, dual-acting therapy has not undergone formal clinical testing. But, we also point out that all of the ingredients are currently approved pharmacopeial articles, having been used for many years with standard nutritional lipid injectable emulsions, since their formal introduction into clinical practice in 1961 (Wretlind, 1981). The potential clinical benefits to patients with CRS of an injectable emulsion of an idealized formulation that delivers precise pharmacological doses and includes the provision of the key substrates, EPA and DHA that mitigates the severe and acute inflammatory response would be desirable. Furthermore, this formulation also provides the same substrates for SPMs that actively foster resolution of inflammation by enhancing removal of apoptotic cellular debris within the TME, which could also be beneficial.

With the current availability of the key pharmaceutical ingredients to produce such a formulation, and the absence of such a product on the market that might be effective when administered in conjunction with CAR T cell therapy, this innovation might be useful in the absence of truly efficacious pharmacotherapy for CRS. Finally, given the aforementioned costs associated with CAR T cell therapy (Sainatham et al., 2024), it may be possible that prophylactic administration of safe amounts of omega-3 fatty acids as described in this review, may allow for more cost-effective treatment in the ambulatory care setting.
